# Shuffling the Neutral Drift of Unspecific Peroxygenase in Saccharomyces cerevisiae

**DOI:** 10.1128/AEM.00808-18

**Published:** 2018-07-17

**Authors:** Javier Martin-Diaz, Carmen Paret, Eva García-Ruiz, Patricia Molina-Espeja, Miguel Alcalde

**Affiliations:** a Department of Biocatalysis, Institute of Catalysis, CSIC, Madrid, Spain; b Manchester Institute of Biotechnology, The University of Manchester, Manchester, United Kingdom; University of Minnesota

**Keywords:** peroxygenases, neutral genetic drift, in vivo DNA shuffling, Saccharomyces cerevisiae, directed evolution

## Abstract

Unspecific peroxygenase (UPO) is a highly promiscuous biocatalyst, and its selective mono(per)oxygenase activity makes it useful for many synthetic chemistry applications. Among the broad repertory of library creation methods for directed enzyme evolution, genetic drift allows neutral mutations to be accumulated gradually within a polymorphic network of variants. In this study, we conducted a campaign of genetic drift with UPO in Saccharomyces cerevisiae, so that neutral mutations were simply added and recombined *in vivo*. With low mutational loading and an activity threshold of 45% of the parent's native function, mutant libraries enriched in folded active UPO variants were generated. After only eight rounds of genetic drift and DNA shuffling, we identified an ensemble of 25 neutrally evolved variants with changes in peroxidative and peroxygenative activities, kinetic thermostability, and enhanced tolerance to organic solvents. With an average of 4.6 substitutions introduced per clone, neutral mutations covered approximately 10% of the protein sequence. Accordingly, this study opens new avenues for UPO design by bringing together neutral genetic drift and DNA recombination *in vivo*.

**IMPORTANCE** Fungal peroxygenases resemble the peroxide shunt pathway of cytochrome P450 monoxygenases, performing selective oxyfunctionalizations of unactivated C-H bonds in a broad range of organic compounds. In this study, we combined neutral genetic drift and *in vivo* DNA shuffling to generate highly functional peroxygenase mutant libraries. The panel of neutrally evolved peroxygenases showed different activity profiles for peroxygenative substrates and improved stability with respect to temperature and the presence of organic cosolvents, making the enzymes valuable blueprints for emerging evolution campaigns. This association of DNA recombination and neutral drift is paving the way for future work in peroxygenase engineering and, from a more general perspective, to any other enzyme system heterologously expressed in S. cerevisiae.

## Abstract (precis)

Fungal peroxygenases resemble the peroxide shunt pathway of cytochrome P450 monoxygenases, performing selective oxyfunctionalizations of unactivated C-H bonds in a broad range of organic compounds. In this study, we combined neutral genetic drift and *in vivo* DNA shuffling to generate highly functional peroxygenase mutant libraries. The panel of neutrally evolved peroxygenases showed different activity profiles for peroxygenative substrates and improved stability with respect to temperature and the presence of organic cosolvents, making the enzymes valuable blueprints for emerging evolution campaigns. This association of DNA recombination and neutral drift is paving the way for future work in peroxygenase engineering and, from a more general perspective, to any other enzyme system heterologously expressed in S. cerevisiae.

## INTRODUCTION

Fungal unspecific peroxygenase (UPO) (EC 1.11.2.1) is a new class of extracellular heme-thiolate peroxidase with exclusive mono(per)oxygenase activity that is attracting the attention of the biotechnology community (1, 2). With broad substrate promiscuity, this highly selective and stable biocatalyst inserts oxygen into unactivated C-H bonds at room temperature and at atmospheric pressure, which represented a mere dream to synthetic chemists only a few years ago. Taking advantage of the peroxide shunt pathway of classic cytochrome P450 monooxygenases, UPO can behave as a self-sufficient monooxygenase triggered by H_2_O_2_, which acts as the final electron acceptor and main oxygen source (3). The spectrum of oxyfunctionalization reactions covered by UPO includes brominations, sulfoxidations, *N*-oxidations, aromatic hydroxylations, alkyl hydroxylations, epoxidations, and ether cleavages, which makes us optimistic about the forthcoming applications of this enzyme as an industrial biocatalyst (4). This is especially true when UPO is compared with the well-studied P450s, whose dependence on expensive redox cofactors and auxiliary flavoproteins, together with the oxygen dilemma associated with the production of unproductive oxygen species and their poor stability, has prevented them from becoming the natural replacements for chemical catalysts in dozens of consolidated industrial reactions (5).

In terms of the industrial use of UPO, one of the main issues that remains pending is its oxidative inactivation by H_2_O_2_, which is currently being addressed by developing sophisticated *in situ* H_2_O_2_ supply systems. Such approaches conceptually unify the fields of chemical catalysis, photocatalysis, and biocatalysis, with the aim of updating longstanding industrial processes through the inclusion of *ad hoc* engineered, efficient, and stable UPO variants (6–8). In this regard, advances in UPO design have recently been achieved through directed evolution, addressing aspects from its heterologous functional expression in yeast to the synthesis of chemicals and pharmaceutical compounds (9–13, 39). These evolutionary enterprises have been carried out by adaptive evolution, i.e., the construction and exploration of mutant libraries to find the best-suited clones according to well-defined selection criteria (14, 15). Unlike traditional adaptive evolution, in which screening efforts focus on the selection of the fittest, directed evolution by neutral genetic drift pursues the gradual accumulation of neutral mutations within a population of variants with similar phenotypes but different genotypes (16). Conversely, iterative rounds of random mutation coupled to selective pressure to maintain the native enzyme function produce polymorphic networks enriched in functional mutants, while detrimental mutations are purged in a process also referred to as “purifying selection” (17). Neutral drift is commonly used to unmask hidden properties, such as latent or promiscuous activities or stability, as demonstrated in studies on P450s, lactonases, β-lactamases, phosphotriesterases, polysialyltransferases, and β-glucuronidases, among other examples (18–26). However, the accumulation of neutral mutations is not straightforward, in part because the activity cutoff employed to maintain the wild-type function favors contamination of the clones selected with parental sequences. Given that ∼45% of mutant libraries generated by low mutational loading are waste, mostly due to the presence of the parental type (27), the search for neutral variants by genetic drift requires substantial experimental input unless ultra-high-throughput screening or genetic selection procedures are available. Indeed, the average numbers of generations in a campaign of neutral drift range from ∼15 to 25 (15, 16, 28). Although the potential to use DNA recombination in genetic drift experiments has long been suggested, all of the studies reported to date have lacked an efficient recombination system that enables neutral mutations to be simultaneously recombined between homologues in each round of neutral evolution.

In the current work, we have prepared a genetic drift protocol that allows neutral mutations to be introduced in conjunction with simultaneous *in vivo* recombination. The “drifted” UPO libraries expressed in Saccharomyces cerevisiae were analyzed in terms of their substrate promiscuity, as well as their stability against temperature and the presence of organic solvents. An ensemble of 25 neutral homologues were subjected to preliminary characterization, and the most promising variants were purified to homogeneity and studied biochemically.

## RESULTS AND DISCUSSION

### Departure point and protocol for shuffling drifted libraries in S. cerevisiae.

**FIG 1:**
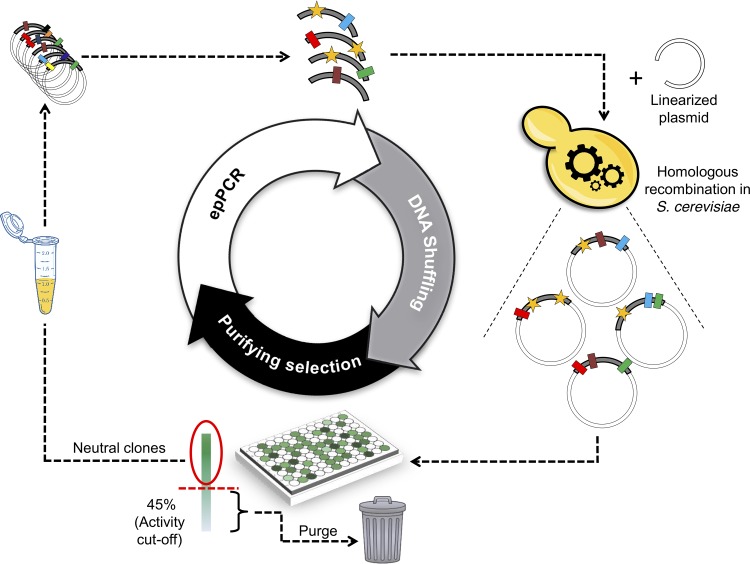
One-pot strategy for neutral drift and *in vivo* DNA shuffling. The epPCR library, with a mutational load of 1 to 3 mutations/kb, was transformed into S. cerevisiae along with the linearized vector. To foster DNA shuffling and cloning *in vivo*, 50-bp overlapping stretches flanking each PCR product, homologous to the ends of the linearized vector, were included. Clones with at least 45% activity with respect to the parent PaDa-I were considered neutral, and their corresponding plasmids were isolated, mixed, and used as the parental type for a new round of neutral drift and DNA shuffling. Rectangles, neutral mutations maintained; stars, new mutations.

As the point of departure for this study, we used a secretion mutant evolved from Agrocybe aegerita UPO for its heterologous functional expression in yeasts, PaDa-I. This variant harbors nine mutations that yield abundant expression in S. cerevisiae and Pichia pastoris, i.e., F12Y-A14V-R15G-A21D-V57A-L67F-V75I-I248V-F311L (the underlined mutations lie in the signal peptide) (9, 10). Performing directed evolution in S. cerevisiae offers many advantages in terms of library creation (29, 30). Given its high frequency of homologous DNA recombination, transformed genes with identities as low as 50% are rapidly shuffled *in vivo* without the need for cleavage sites or DNases. Thus, to set out the genetic drift campaign, we wired each round of random mutation to *in vivo* DNA shuffling by designing 50-bp flanking overhangs between the N and C termini of the error-prone PCR (epPCR) products and the linearized vector (Fig. 1). Using this strategy, neutral unfragmented homologues were freely recombined, whereas the full autonomously replicating plasmid was repaired in just a single transformation step. A mutational load of 1 to 3 substitutions per kilobase was chosen because higher mutation frequencies would increase the number of inactive clones and jeopardize the diversity in the drifted library.

Although the evolutionary lineage of natural UPO remains unclear, the convergence of peroxidative activity (1-electron oxidation reactions) and peroxygenative activity (oxygen transfer by 2-electron oxidations) within the same enzyme provides insight into its original native function. Considering UPO as the first truly natural peroxygenase implies that this enzyme is the missing catalytic link between heme-thiolate enzymes and classic peroxidases (3). The hybrid catalytic mechanism employed by UPO suggests that peroxygenative activity could plausibly have evolved from an ancestral peroxidative activity. Therefore, to maintain the protein's original function and structure while gradually accumulating neutral mutations, we imposed the constraint that UPO variants had to oxidize a peroxidative substrate and to exceed a threshold of 45% of the parental activity. This activity threshold represented a compromise between improved stability and the exploration of promiscuous activities. Indeed, stringent cutoff values (∼75%) are used to improve stability, whereas more relaxed ones (∼30%) are established for latent activities (16). Given that the physiological function of UPO is still unknown (among the possible roles in nature, the synthesis of metabolites, detoxification processes, and humus and lignin degradation have been proposed), we chose 2,2′-azino-bis(3-ethylbenzothiazoline-6-sulfonic acid) (ABTS) as a surrogate peroxidative substrate for use during purifying selection. ABTS is commonly used to screen heme-containing peroxidase libraries, and it offers excellent sensitivity limits, low coefficients of variance, and minimal interactions with the yeast culture broth (9).

### Neutral drift campaign.

**FIG 2:**
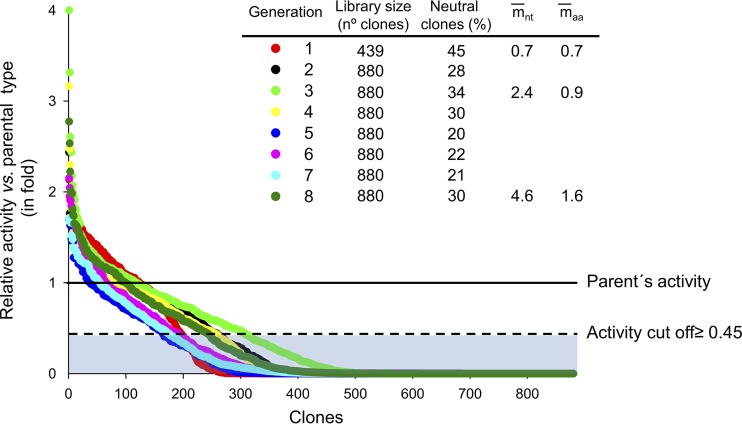
Neutral drift-DNA shuffling landscapes from generations 1 to 8. The activities of the clones are plotted in descending order; the solid line shows the activity of the parent PaDa-I, and the dashed line represents the activity threshold, with purged clones within the blue region. Clones satisfying the activity threshold were considered neutral and were used as parental variants in subsequent rounds. m̄_nt_, average nucleotide mutations; m̄_aa_, average amino acid mutations.

With the aforementioned premises, the PaDa-I variant was subjected to eight rounds of neutral drift combined with *in vivo* shuffling. Although the same mutational load was maintained throughout the genetic drift experiment, the percentage of selected neutral clones that conformed to the activity threshold in each round fluctuated slightly, as a consequence of the recombination in yeast and the gradual accumulation of neutral mutations (with 45% and 30% functional clones in rounds 1 and 8, respectively) (Fig. 2). In generation 3, we established a midcheckpoint for the drifted library by selecting 30 neutral clones at random (using Python [random number generator] software). We first monitored the accumulation of mutations by sequencing the selected variants. Of the 30 clones, 13 incorporated amino acid substitutions in the mature UPO, with an average mutational rate of 2.4 nucleotide mutations per clone. In addition, 12 clones contained only silent mutations, which may have an important role in future adaptation processes, and 5 clones were of the parental type. The 13 neutral variants with amino acid substitutions were produced and subjected to preliminary characterization. The majority of the mutations were situated at the surface of the protein, far from the relevant catalytic sites (see Fig. S1 in the supplemental material). When kinetic thermostability was estimated by measuring the temperature at which the enzyme lost 50% of its activity after a 10-min incubation (*T*_50_) for the variants, 3 mutants showed an ∼2°C improvement over the parental enzyme (clone 4, V298A; clone 33, K302Q; clone 39, V247A) (Fig. S2A). In terms of activity, the initial turnover rates for the peroxidative substrates ABTS and 2,6-dimethoxyphenol (DMP) and the peroxygenative substrates 5-nitro-1,3-benzodioxole (NBD) and naphthalene were assessed, with some mild deviations being detected e.g., the activity of variant 4 with ABTS, DMP, and NBD improved around 1.5-fold (Fig. S2B). In light of these data, we moved forward with the neutral drift-DNA shuffling campaign until eight rounds were completed (∼7,000 clones screened).

**FIG 3:**
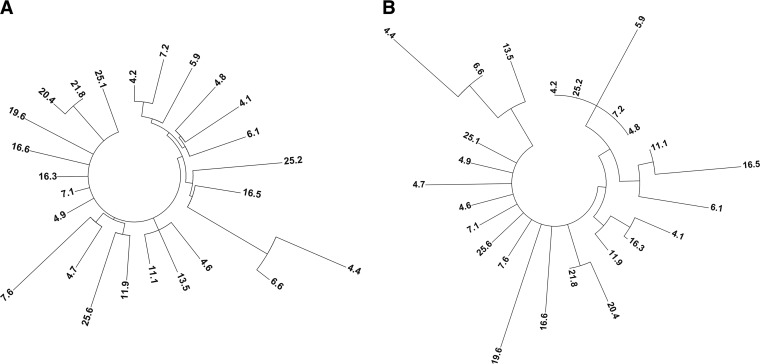
Cladograms of neutrally evolved UPOs. The trees (predicted by ClustalX 2.1 and represented by Mega 6) show the connections between 25 UPO homologues from generation 8 created by neutral drift and DNA shuffling. (A) Cladogram constructed from nucleotide substitutions (including silent mutations). (B) Cladogram constructed from amino acid substitutions (see Table S1 in the supplemental material).

**FIG 4:**
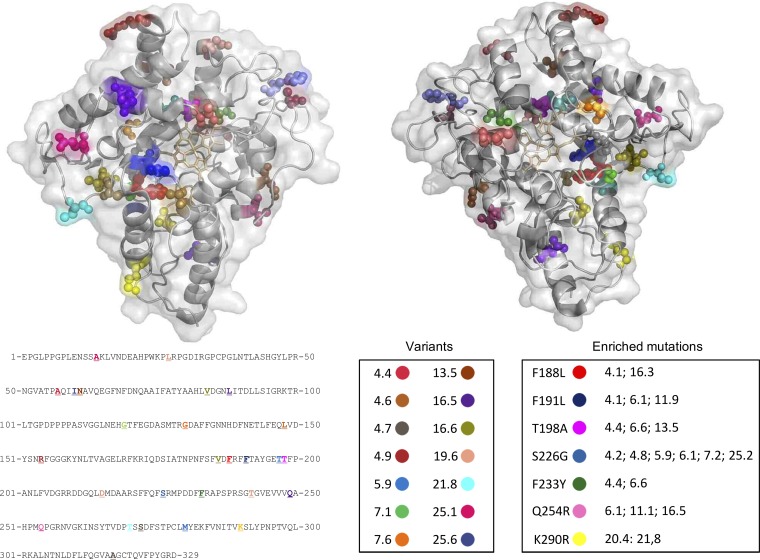
Mutations of the neutrally evolved UPOs. Mutations of the 25 variants extracted from generation 8 are highlighted in different colors and related to the clone number (front and back perspectives). Enriched mutations appear in several mutants (see Table S1 in the supplemental material). Mutations are mapped in the A. aegerita UPO crystal structure (PDB accession number 2YOR).

Of the 191 neutral clones obtained in generation 8, 25 were produced and their culture supernatants were used for preliminary characterization. The broad sequence diversity of these neutrally evolved homologues was evident in the phylogenetic trees derived from nucleotide and amino acid substitutions (Fig. 3). On average, we identified 4.6 nucleotide mutations per clone (115 mutations in total), of which 40 were nonsynonymous mutations over 27 positions (almost 10% of the protein sequence) (Table S1). Eighty percent of the mutations were situated at the surface of the protein, with only 2 mutations in the catalytic center (Fig. 4), whereas we found 25% enrichment in mutations (i.e., appearing in more than 1 sequence). It is highly likely that many of the enriched mutations were actually consensus-ancestor mutations, as also observed in other neutral drift campaigns (21), given their locations (the majority of them were at the surface of the protein structure, far from the catalytic pocket) and their thermostabilizing effects; however, the influence of DNA recombination on such enrichment cannot be ruled out. Unfortunately, UPO is a relatively novel protein, and the lack of sufficient reliable sequences (to date, only 4 UPOs, from Agrocybe aegerita [31], Coprinellus radians [32], Marasmius rotula [33], and Chaetomium globosum [34], have been characterized) hampered the performance of a solid multiple-sequence alignment to determine the possible consensus-ancestor origins of neutral mutations. The increasing number of hypothetical UPO sequences deposited in genomic databases, along with the efforts of our laboratory and others in expressing new UPO sequences, will facilitate consensus design and phylogenetic analysis aimed at finding stabilizing consensus-ancestor mutations or even resurrecting ancestral nodes for UPO proteins in the near future (12).

**FIG 5:**
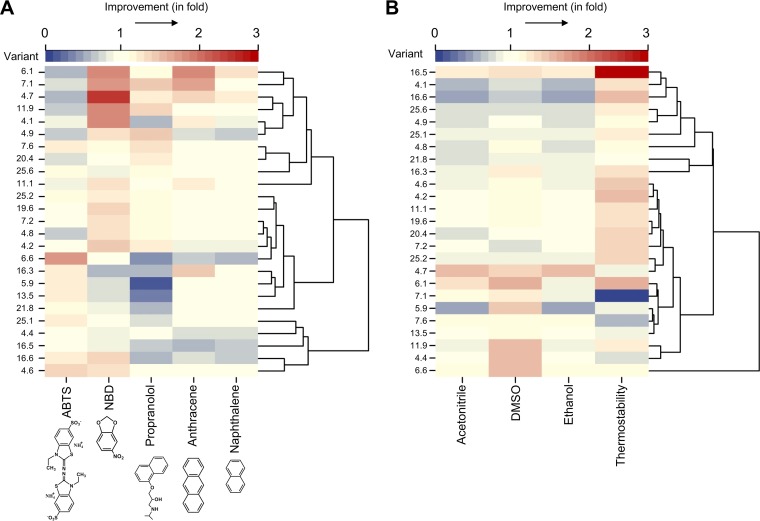
Range of activities and stabilities of neutrally evolved UPOs. Heat maps of activities (A) and stabilities (B) show the fold improvements, relative to the parental type, for the 25 neutrally evolved UPO variants from generation 8. In both maps, the variants were hierarchically organized into dendrograms, according to their activity (A) and their tolerance to temperature and cosolvents (B), using the R-studio program and the package ape to arrange the different variants in clusters (38). Activity and stability measurements were made in triplicate from supernatant preparations, as described in Materials and Methods.

The substrate promiscuity of the neutral variants was first analyzed with a panel of peroxygenative substrates, including NBD, propranolol, anthracene, and naphthalene, as well as with the peroxidative substrate ABTS. A heat map enabled the neutral variants to be arranged into hierarchical clusters according to the improved activity with all five substrates, relative to the neutral evolution parent PaDa-I (Fig. 5A). The change in activity, determined as the ratio of the variant's activity for each substrate to that of the parent PaDa-I, allowed us to easily sort the neutral variants according to their activity preferences while discriminating among possible secretion mutants. The lack of correlation between the activities for the five substrates addressed the absence of secretion mutants within the set of neutral clones analyzed, which was confirmed by SDS-PAGE analysis (Fig. S3). There was a modest increase or decrease in the oxidation of ABTS for the majority of the neutral variants, although all retained at least 45% of the activity of PaDa-I (the minimum requirement for selection). The average improvement/reduction ratio of neutral variants for oxidization of ABTS, with respect to the activity of PaDa-I, was close to 1 (ratio of 0.95 ± 0.3). More pronounced changes in the latent activities for peroxygenative substrates were observed, compared to the activities detected for ABTS. For example, many of the variants had ∼2-fold improved activity for the dealkylation of NBD, with the order of preference for the oxidation of peroxygenative substrates being as follows: NBD > propranolol > anthracene > naphthalene. The robustness of the variants, in terms of tolerance to high temperatures and organic solvents, was also monitored; as evident with substrate preferences, substantial changes were detected, although a clear relationship between thermostability and tolerance to cosolvents could not be established (Fig. 5B). Approximately one-half of the variants had improved thermostability, while the resistance to cosolvents mostly followed the pattern dimethyl sulfoxide (DMSO) > acetonitrile > ethanol.

### Biochemical characterization of purified neutral variants.

To assess the detected changes in stability and activity in more detail, several neutral variants (i.e., 6.1 [F191L-S226G-Q254R], 7.1 [G119S], 4.7 [S272P-A317D], and 16.5 [L88P-Q249R-Q254R]) were produced on a larger scale and purified to homogeneity (Reinheitszahl [R_Z_] value [*A*_418_/*A*_280_] of ∼2) (Fig. S4A to C). In terms of stability, the 16.5 variant was more thermostable, improving the half-life (*t*_1/2_) of the parent PaDa-I by 34 min, while the 6.1 variant duplicated the parental value (*t*_1/2_ was defined as the time required to lose 50% of the activity with incubation of the enzyme at 63°C) (Table 1; also see Fig. S5A in the supplemental material). Strikingly, both the 6.1 and 4.7 variants notably increased their tolerance to organic solvents, especially to acetonitrile, with *C*_50_ values of ∼20%, compared to 7% for the parent PaDa-I (the *C*_50_ was defined as the concentration of cosolvent, expressed as a percentage [by volume], at which the enzyme lost one-half of its activity) (Table 1; also see Fig. S5B to D). This tolerance was also evident to a lesser extent for acetone, methanol, and DMSO but not for ethanol. The kinetic parameters of the variants were assessed with a panel of peroxidative (ABTS and DMP) and peroxygenative (veratryl alcohol, benzyl alcohol, NBD, naphthalene, and propranolol) substrates (Table 2). The peroxygenative/peroxidative ratio plays a key role in the aromatic hydroxylation reactions performed by UPO; the products of peroxygenative activity on aromatic compounds (phenolics) become peroxidative substrates, thus being oxidized by UPO into phenoxyl radicals and jeopardizing the final reaction yields. Some of the neutrally evolved variants in our study showed slight changes in the peroxygenative/peroxidative ratio that were dependent on the substrate tested. In this regard, recent findings (11, 13, 39; unpublished data) indicate that the peroxygenative/peroxidative ratio is mostly dependent on the nature of the targeted substrates, which defines the access to the heme channel and the residence time at the binding site for proper oxygenation/oxidation, rather than the existence of catalytic radical-forming residues at the protein surface to work through a long-range electron-transfer pathway to the heme, as described for ligninolytic peroxidases (35). The variants with the greatest thermostabilities (variants 16.5 and 6.1) displayed overall decreases in catalytic efficiencies (*k*_cat_/*K_m_*), which may be connected to a putative tradeoff between activity and stability that can be circumvented by different engineering strategies (reference 36 and references cited therein). The most notable improvements in activity were detected for variants 7.1 and 4.7; while variant 7.1 showed enhancements of up to ∼1.5-fold in *k*_cat_/*K_m_* values for NBD, naphthalene, and DMP, variant 4.7 had 1.7- and 1.5-fold enhanced catalytic efficiencies for NBD and propranolol, respectively. The latter is of special pharmaceutical interest, since this compound is a widely used beta blocker that UPO can convert into the equipotent human drug metabolite 5-OH′-propranolol (39).

**TABLE 1:** Kinetic thermostability and activity in organic solvents^*a*^

Parameter	PaDa-I	Variant 16.5	Variant 6.1	Variant 7.1	Variant 4.7
Thermostability *t*_1/2_ (min)	8.7	43	16.6	2.9	8.7
*C*_50_ (%)					
Acetonitrile	7.0	7.2	17.5	8.8	20.3
DMSO	2.0	1.7	2.8	1.3	2.3
Ethanol	1.0	1.0	1.0	1.0	1.0
Methanol	8.4	8.8	9.6	7.8	9.7
Acetone	10.0	11.6	13.2	8.1	13.1

a Values were calculated from the *t*_1/2_ and *C*_50_ plots of Fig. S5 in the supplemental material.

**TABLE 2:** Kinetic parameters for PaDa-I and neutrally evolved UPO variants^*a*^

Substrate and kinetic parameter	PaDa-I	Variant 16.5	Variant 6.1	Variant 7.1	Variant 4.7
ABTS					
*K_m_* (mM)	0.067 ± 0.009	0.034 ± 0.003	0.052 ± 0.03	0.09 ± 0.02	0.09 ± 0.01
*k*_cat_ (s^−1^)	670 ± 37	513 ± 12	370 ± 7	322 ± 43	246 ± 15
*k*_cat_*/K_m_* (mM^−1^ s^−1^)	10,224 ± 1,026	15,057 ± 2,037	7,073 ± 285	3,504 ± 710	2,585 ± 244
DMP					
*K_m_* (mM)	0.088 ± 0.003	0.09 ± 0.01	0.29 ± 0.02	0.046 ± 0.006	0.21 ± 0.01
*k*_cat_ (s^−1^)	167 ± 2	76 ± 8	162 ± 5	108 ± 4	264 ± 3
*k*_cat_*/K_m_* (mM^−1^ s^−1^)	1,899 ± 51	777 ± 88	543 ± 27	2,396 ± 259	1,205 ± 25
NBD					
*K_m_* (mM)	0.66 ± 0.21	1.77 ± 0.51	0.65 ± 0.2	0.20 ± 0.03	0.19 ± 0.07
*k*_cat_ (s^−1^)	303 ± 40	160 ± 26	170 ± 20	126 ± 4	131 ± 8
*k*_cat_*/K_m_* (mM^−1^ s^−1^)	460 ± 108	90 ± 11	262 ± 50	629 ± 77	710 ± 222
Propranolol					
*K_m_* (mM)	2.1 ± 0.1	2.5 ± 0.2	5.7 ± 2.1	2.1 ± 0.2	0.61 ± 0.09
*k*_cat_ (s^−1^)	186 ± 6	25 ± 1	255 ± 68	167 ± 10	81 ± 4
*k*_cat_*/K_m_* (mM^−1^ s^−1^)	90 ± 3	10.0 ± 0.5	44 ± 5	78 ± 4	131 ± 13
Naphthalene					
*K_m_* (mM)	0.38 ± 0.09	0.49 ± 0.09	0.59 ± 0.07	0.19 ± 0.05	0.48 ± 0.05
*k*_cat_ (s^−1^)	162 ± 14	119 ± 9	89 ± 4	97 ± 7	127 ± 5
*k*_cat_*/K_m_* (mM^−1^ s^−1^)	421 ± 69	243 ± 31	150 ± 10	520 ± 116	264 ± 18
Veratryl alcohol					
*K_m_* (mM)	12 ± 0.8	10 ± 1	9 ± 3	7 ± 1	20 ± 3
*k*_cat_ (s^−1^)	256 ± 8	141 ± 6	56 ± 7	107 ± 5	220 ± 22
*k*_cat_*/K_m_* (mM^−1^ s^−1^)	21 ± 1	13 ± 0.8	6 ± 1	16 ± 2	11 ± 1
Benzyl alcohol					
*K_m_* (mM)	2.3 ± 0.3	2.5 ± 0.1	11 ± 2	2.3 ± 0.3	4.5 ± 0.3
*k*_cat_ (s^−1^)	630 ± 26	506 ± 9	426 ± 50	282 ± 13	558 ± 17
*k*_cat_*/K_m_* (mM^−1^ s^−1^)	271 ± 26	204 ± 8	39 ± 5	121 ± 13	124 ± 7

a For each substrate, reactions were performed in triplicate, with monitoring of the increases in absorption for ABTS (ε_418_ = 36,000 M^−1^ cm^−1^), NBD (ε_425_ = 9,700 M^−1^ cm^−1^), DMP (ε_469_ = 27,500 M^−1^ cm^−1^), propranolol (ε_325_ = 1,996 M^−1^ cm^−1^), naphthalene (ε_303_ = 2,010 M^−1^ cm^−1^), benzyl alcohol (ε_280_ = 1,400 M^−1^ cm^−1^), and veratryl alcohol (ε_310_ = 9,300 M^−1^ cm^−1^). Further details are presented in Materials and Methods.

### Conclusions.

Neutral genetic drift is a powerful tool to modify the substrate promiscuity and stability of enzymes, whereas DNA shuffling is a long-established recombination method to unify beneficial mutations from different parents and/or to purge detrimental ones. In this work, we describe a “one-pot” approach that brings together genetic drift and DNA shuffling in S. cerevisiae in order to generate highly functional UPO libraries. Indeed, the accumulation of neutral mutations and their simultaneous recombination *in vivo* helped speed up the genetic drift process while removing destabilizing mutations in a drive toward more evolvable drifted libraries. The less stringent activity threshold established during screening allowed both the stability and activity of the UPO variants to be controlled. Given that many of the industrial reactions that UPO can perform take place under harsh conditions, some of the neutrally evolved UPOs from this study represent promising departure points for further engineering toward greater tolerance to cosolvents or stronger thermostabilities. Moreover, some modifications found in the range of activities, such as transformation to the promiscuous substrate propranolol, open up avenues to design highly efficient UPO variants for the synthesis of human drug metabolites, which are important compounds in pharmacokinetic and pharmacodynamics studies.

## MATERIALS AND METHODS

### Materials.

The Agrocybe aegerita UPO secretion mutant (PaDa-I) was obtained as described elsewhere (9). The expression shuttle vector pJRoC30, with uracil auxotrophy and an ampicillin marker for selection, came from the California Institute of Technology (USA). ABTS, DMP, veratryl alcohol, benzyl alcohol, Tween-20, hemoglobin from bovine blood, ascorbic acid, anthracene, *Taq* DNA polymerase, and a yeast transformation kit were purchased from Sigma-Aldrich (Madrid, Spain). NBD was acquired from TCI America (Portland, OR, USA). Naphthalene and dl-propranolol hydrochloride were obtained from Acros Organics (Geel, Belgium). S. cerevisiae strain BJ5465 was from LGC Promochem (Barcelona, Spain), whereas Escherichia coli XL2-Blue competent cells were from Stratagene (La Jolla, CA, USA). The Zymoprep yeast plasmid miniprep kit and Zymoclean gel DNA recovery kit were from Zymo Research (Orange, CA, USA). The NucleoSpin plasmid kit was purchased from Macherey-Nagel (Düren, Germany). The restriction enzymes BamHI and XhoI were from New England BioLabs (Hertfordshire, UK). Oligonucleotides were synthesized by Metabion (Bayern, Germany). All chemicals were of reagent-grade purity.

### Culture media.

Minimal medium, synthetic complete (SC) dropout plates, and Luria-Bertani (LB) medium were prepared as reported elsewhere (9). Selective expression medium contained 100 ml of filtered 6.7% yeast nitrogen base, 100 ml of filtered 19.2 g/liter yeast synthetic dropout medium without uracil, 100 ml of filtered 20% galactose, 67 ml of filtered 1 M KH_2_PO_4_ buffer (pH 6.0), 22 ml of filtered 0.1 M MgSO_4_, 34.8 ml of absolute ethanol, 1 ml of filtered 25 g/liter chloramphenicol, and double-distilled water up to 1 liter. Hemoglobin expression medium included 712.5 ml of yeast-peptone medium (1.55×), 66 ml of filtered 1 M KH_2_PO_4_ buffer (pH 6.0), 110 ml of 20% filtered galactose, 22 ml of filtered MgSO_4_, 31.5 ml of absolute ethanol, 16.5 ml of filtered 20 g/liter hemoglobin, 1.1 ml of filtered 25 g/liter chloramphenicol, and double-distilled water up to 1 liter.

### Mutant library creation.

Eight rounds of neutral drift and DNA shuffling were performed. pJRoC30 was linearized with BamHI and XhoI. The linearized vector was cleaned, concentrated, loaded onto a low-melting-point preparative agarose gel, and purified using the Zymoclean gel DNA recovery kit.

### (i) Error-prone PCR.

Except for the first round of neutral drift (in which PaDa-I was the parental type), the collection of plasmids satisfying the activity threshold (see below) was subjected to epPCR with *Taq* DNA polymerase in the presence of MnCl_2_ (mutational load, 1 to 3 mutations/kb). Primers used for amplifications were RMLN (sense; 5′-CCTCTATACTTTAACGTCAAGG-3′) and RMLC (antisense; 5′-GGGAGGGCGTGAATGTAAGC-3′). The epPCR was carried out in a final volume of 50 μl containing 3% DMSO, 90 nM RMLN, 90 nM RMLC, 0.3 mM deoxynucleoside triphosphates (dNTPs) (0.075 mM each), 0.01 mM MnCl_2_, 1.5 mM MgCl_2_, 0.05 U/μl *Taq* DNA polymerase, and 0.14 ng/μl of the corresponding templates. The epPCR was performed on a gradient thermocycler (Mycycler; Bio-Rad, USA), using the following parameters: 95°C for 2 min (1 cycle); 94°C for 45 s, 55°C for 30 s, and 74°C for 90 s (28 cycles); and 74°C for 10 min (1 cycle). PCR products were purified using the Zymoclean gel DNA recovery kit.

### (ii) *In vivo* DNA shuffling.

Two hundred nanograms of epPCR product was mixed with 100 ng of the linearized vector and transformed into competent S. cerevisiae cells using the yeast transformation kit. Inserts and linearized plasmid shared 50 bp of homology, to allow recombination and *in vivo* cloning by the yeast. Transformed cells were plated on SC dropout plates and incubated for 3 days at 30°C.

### High-throughput screening.

Individual colonies were picked and cultured in 96-well plates containing 210 μl of selective expression medium per well. In each plate, column 6 was inoculated with parent PaDa-I and well H1 (containing minimal medium supplemented with uracil) was inoculated with untransformed S. cerevisiae as a negative control. Plates were incubated at 30°C, at 230 rpm, in 80% relative humidity (Minitron-INFORS, Switzerland). After 72 h, plates were centrifuged (Eppendorf 5810R centrifuge; Eppendorf, Germany) for 10 min at 3,500 rpm, at 4°C. Twenty microliters of supernatant was transferred to new plates at a robotic liquid-handling station (Freedom EVO 100 base; TECAN Schweiz AG, Switzerland), and 180 μl of a reaction mixture (100 mM sodium phosphate/citrate buffer [pH 4.4], 0.3 mM ABTS, and 2 mM H_2_O_2_) was added to each plate with the help of a pipetting robot (Multidrop Combi reagent dispenser; Thermo Scientific, USA). The plates were briefly stirred, and the absorbance at 418 nm (ε_ABTS_ = 36,000 M^−1^ cm^−1^) was measured in kinetic mode with a plate reader (SPECTRAMax Plus 384; Molecular Devices, USA). The values obtained were normalized to those for the corresponding parental type in each plate (9).

For purifying selection, the cutoff value for UPO activity was set at 45% of the parental activity (clones with activity below that threshold were purged). Using the high-throughput screening method described above, the selected clones (20 μl each from resuspended cell pellets) were pooled and subjected to plasmid extraction using the Zymoprep yeast plasmid miniprep kit. To remove impurities and to enhance the yield, the resulting mixed DNA product was transformed into E. coli XL2-Blue cells, and the cells were plated on a LB agar plate with ampicillin and grown overnight at 37°C. Transformed colonies were scratched from the LB agar plate and inoculated in 2 ml of LB liquid medium. Mutant library plasmid extraction was performed using the NucleoSpin plasmid kit. The purified mixture was used as the template for a new round of epPCR and DNA shuffling, as described above.

### Production of neutral variants.

For each selected neutral clone, cell pellets were resuspended by pipetting up and down and stirring. Twenty microliters of resuspended mixture was transferred to 3 ml of minimal medium. After 48 h at 30°C and 220 rpm, plasmids were extracted with the Zymoprep kit. E. coli XL2-Blue cells were transformed with the Zymoprep product, plated on LB agar plates with ampicillin, and grown overnight at 37°C. Single colonies were inoculated in 5 ml of LB liquid medium with ampicillin and were grown overnight at 37°C. Plasmids were extracted and transformed into S. cerevisiae cells, and the cells were plated on SC dropout plates. After 3 days at 30°C, single colonies were inoculated in 5 ml of minimal medium and incubated for 48 h at 30°C, at 220 rpm. Clones were refreshed in a final volume of 5 ml of minimal medium with an optical density at 600 nm (OD_600_) of 0.3. After 6 to 8 h of growth (OD_600_ of 1 to 1.5), 9 ml of hemoglobin expression medium was inoculated with 1 ml of preculture and incubated for 48 h at 30°C, at 250 rpm, in a 100-ml flask. Growth and expression were monitored by measuring the OD_600_ of the cultures and the activity against ABTS, as described below, until the stationary phase was reached. Cells were removed by centrifugation at 3,500 rpm for 15 min at 4°C, and the supernatant was saved for activity and stability assays.

### Activity and stability assays. (i) ABTS.

Twenty microliters of supernatant was mixed with 180 μl of 100 mM sodium phosphate/citrate buffer (pH 4.4) containing 0.3 mM ABTS and 2 mM H_2_O_2_. The plates were briefly stirred, and the absorbance was measured at 418 nm (ε_418_ = 36,000 M^−1^ cm^−1^) (9).

### (ii) NBD.

Twenty microliters of supernatant was mixed with 180 μl of 100 mM sodium phosphate buffer (pH 7.0) containing 1 mM NBD (final concentration of acetonitrile, 15%) and 1 mM H_2_O_2_. The plates were briefly stirred, and the absorbance was measured at 425 nm (ε_425_ = 9,700 M^−1^ cm^−1^) (9).

### (iii) Naphthalene.

Twenty microliters of supernatant was mixed with 180 μl of 100 mM potassium phosphate buffer (pH 7.0) containing 0.5 mM naphthalene (final concentration of acetonitrile, 10%) and 1 mM H_2_O_2_. After a reaction time of 10 min, 20 μl of Fast Red TR Salt hemi(zinc chloride) salt was added to each well and the plates were incubated at room temperature until a red color developed. The absorption was measured at 510 nm (ε_510_ = 4,700 M^−1^ cm^−1^) (11).

### (iv) Propranolol.

Forty microliters of supernatant was mixed with 180 μl of 100 mM sodium phosphate buffer (pH 7.0) containing 5 mM propranolol, 2 mM H_2_O_2_, and 4 mM ascorbic acid. After a reaction time of 60 min, the sample was subjected to the 4-aminoantipyrine (4-AAP) assay, with minor modifications (39). Plates were briefly stirred, and the absorption at 530 nm was recorded.

### (v) Anthracene.

Four hundred microliters of supernatant was mixed with 600 μl of 20 mM potassium phosphate buffer (pH 7.0) containing 1 mM anthracene (final concentration of acetonitrile, 20%), 1% Tween-20, and 1 mM H_2_O_2_. After 1 h, reactions were stopped by incubation for 10 min at 90°C. Activities were analyzed by reverse-phase high-performance liquid chromatography (HPLC) with equipment consisting of a tertiary pump (Varian/Agilent Technologies, USA) coupled to an autosampler (Merck Millipore, USA) and an ACE C_18_ pentafluorophenyl column (15 cm by 4.6 mm) at 40°C. Detection was performed at 355 nm with a photometric diode array (PDA) detector (Variant/Agilent Technologies). The mobile phase was methanol (80%) and double-distilled water (20%), at a flow rate of 0.8 ml min^−1^.

### (vi) Activity in organic solvents.

The relative activities in organic solvents were assessed with the ABTS assay described above (100 mM sodium phosphate/citrate buffer [pH 4.4], 0.3 mM ABTS, and 2 mM H_2_O_2_), supplementing the reaction mixture with the corresponding concentration of organic solvent (12% acetonitrile, 6% DMSO, or 3% ethanol) and appropriate dilutions of supernatants. Tolerance in organic solvent (i.e., retained activity with cosolvents) was defined as the ratio of the activity in the presence of organic solvent to that in the absence of organic solvent, expressed as fold change from the parental type.

### (vii) Kinetic thermostability.

Appropriate dilutions of supernatants were prepared in 10 mM potassium phosphate buffer (pH 7.0) in such a way that 20-μl aliquots gave rise to a linear response in kinetic mode. Fifty microliters of supernatant was used for each point on a gradient ranging from 30°C to 80°C. This gradient profile was achieved using a thermocycler. After 10 min of incubation, samples were removed and chilled on ice for 10 min. After that, samples of 20 μl were removed and incubated at room temperature for 5 min. Finally, samples were subjected to the same ABTS colorimetric assay as described above for the screening (100 mM sodium phosphate/citrate buffer [pH 4.4], 0.3 mM ABTS, and 2 mM H_2_O_2_). Thermostability values were calculated from the ratios between the residual activities at different temperatures and the initial activity at room temperature. The *T*_50_ value was determined as the transition midpoint of the curve for protein inactivation as a function of temperature, which in our case was defined as the temperature at which the enzyme lost 50% of its activity following incubation for 10 min.

### Purification.

Mutants 6.1, 4.7, 7.1, 16.5, and PaDa-I were produced and purified to homogeneity. A single S. cerevisiae colony from each variant was inoculated in 20 ml of minimal medium and incubated for 48 h at 30°C, at 230 rpm. Clones were refreshed in a final volume of 250 ml of minimal medium, at an OD_600_ of 0.3. After 6 to 8 h of growth (OD_600_ of 1 to 1.5), 900 ml of hemoglobin expression medium was inoculated with 100 ml of preculture and grown for 72 h at 25°C, at 250 rpm. Expression was monitored by measuring the OD_600_ of the cultures and the activity against ABTS, as described above, until the stationary phase was reached. Cells were removed by centrifugation at 6,000 rpm for 30 min at 4°C, and the supernatants were saved for enzyme assays. Supernatants were filtered using a nitrocellulose membrane of 0.45-μm pore size. The supernatants were then concentrated using a Pellicon tangential ultrafiltration system (10-kDa-cutoff membrane; Millipore, USA) and an Amicon stirred ultrafiltration cell (10-kDa-cutoff membrane; Millipore), followed by dialysis against 20 mM sodium citrate buffer (pH 3.3) (buffer A). The samples were filtered and loaded onto two cation-exchange HiTrap SP FF columns in a row, connected to an ÄKTA purifier system (GE Healthcare, UK) and preequilibrated with buffer A. Proteins were eluted with a 60-min linear gradient from 0 to 40% buffer A containing 1 M NaCl. Fractions with ABTS activity were collected, concentrated, dialyzed against 20 mM Tris-HCl buffer (pH 7.8) (buffer B), and loaded onto a BioSuite Q anion-exchange column (Waters, USA) preequilibrated with buffer B. Proteins were eluted with a 40-min linear gradient from 0 to 20% buffer B containing 1 M NaCl. The fractions with UPO activity toward ABTS were collected and dialyzed against 10 mM potassium phosphate buffer (pH 7.0). Samples of pure enzymes were stored at 4°C. The R_Z_ values (*A*_418_/*A*_280_) achieved were ∼2. Throughout the purification protocol, the fractions were analyzed by SDS/PAGE on 12% gels, and the proteins were stained with SeeBand protein staining solution (Gene Bio-Application, Israel). The concentrations of all crude protein extracts were determined using the Bio-Rad protein reagent, with bovine serum albumin (BSA) as the standard.

### Biochemical characterization of purified neutral variants. (i) Kinetic parameters.

Kinetic values were estimated with increasing substrate concentrations; data were fitted to a single rectangular hyperbolic function with a Michaelis-Menten model by using SigmaPlot 10.0, where parameter *a* was equal to *k*_cat_ and parameter *b* was equal to *K*_m_. Kinetic analyses for ABTS were performed in 100 mM sodium citrate/phosphate buffer (pH 4.0) containing 2 mM H_2_O_2_. Kinetic analyses for NBD were performed in 100 mM potassium phosphate buffer (pH 7.0) containing 1 mM H_2_O_2_ in 15% acetonitrile. Kinetic analyses for DMP were carried out in potassium phosphate buffer (pH 7.0) containing 2 mM H_2_O_2_. Kinetic analyses for naphthalene were performed in 100 mM potassium phosphate buffer (pH 7.0) containing 1 mM H_2_O_2_ in 20% acetonitrile. Kinetic analyses for propranolol were performed in potassium phosphate buffer (pH 7.0) containing 2 mM H_2_O_2_ and 4 mM ascorbic acid. Kinetic analyses for benzyl alcohol were performed in potassium phosphate buffer (pH 7.0) containing 2 mM H_2_O_2_. Kinetic analyses for veratryl alcohol were carried out in potassium phosphate buffer (pH 7.0) containing 2 mM H_2_O_2_. For each substrate, reactions were performed in triplicate, with monitoring of the increases in absorption for ABTS (ε_418_ = 36,000 M^−1^ cm^−1^), NBD (ε_425_ = 9,700 M^−1^ cm^−1^), DMP (ε_469_ = 27,500 M^−1^ cm^−1^), propranolol (ε_325_ = 1,996 M^−1^ cm^−1^), naphthalene (ε_303_ = 2,010 M^−1^ cm^−1^), benzyl alcohol (ε_280_ = 1,400 M^−1^ cm^−1^), and veratryl alcohol (ε_310_ = 9,300 M^−1^ cm^−1^).

### (ii) Determination of *C*_50_.

Activity in organic solvents was assessed in kinetic mode using the ABTS assay described above (100 mM sodium phosphate/citrate buffer [pH 4.4], 0.3 mM ABTS, and 2 mM H_2_O_2_), with the corresponding concentrations of cosolvent and appropriate dilutions of enzymes. The *C*_50_ was defined as the concentration of cosolvent (expressed as a percentage [by volume]) at which the enzyme showed 50% of the corresponding activity in buffer.

### (iii) Determination of *t*_1/2_.

Appropriate dilutions of enzymes in 10 mM potassium phosphate buffer (pH 7.0) were incubated at 63°C. Every 5 min, 20-μl aliquots were removed and residual activity was determined in kinetic mode with the ABTS assay described above (100 mM sodium phosphate/citrate buffer [pH 4.4], 0.3 mM ABTS, and 2 mM H_2_O_2_). The half-life (*t*_1/2_) was defined as the time required by the enzyme, after incubation at 63°C, to lose 50% of its initial activity at room temperature.

### DNA sequencing.

UPO genes were sequenced by GATC-Biotech. The primers used were RMLN, apo1secdir (5′-GAGCCAGGATTACCTCCTG-3′), apo1secrev (5′-GGTCATACTGGCGTCGCCTTC-3′), and RMLC.

### Protein modeling.

The mutations introduced by neutral genetic drift were mapped using the crystal structure of native UPO from A. aegerita (PDB accession number 2YOR) at a resolution of 2.1 Å (37). The model was generated and analyzed by the PyMOL molecular visualization system.

## Supplementary Material

Supplemental material
