# Supplementary material for: Shuffling the Neutral Drift of Unspecific Peroxygenase in Saccharomyces cerevisiae
Source: Appl Environ Microbiol. 2018 Jul 17;84(15):e00808-18. doi: 10.1128/AEM.00808-18 (PMC6052263; doi:10.1128/AEM.00808-18)
Supplement: Supplemental material [file supp_84_15_e00808-18__index.html]

Shuffling the Neutral Drift of Unspecific Peroxygenase in Saccharomyces cerevisiae — Supplemental material 

# Shuffling the Neutral Drift of Unspecific Peroxygenase in Saccharomyces cerevisiae

## Supplemental material

- Supplemental file 1 -

  Mutation of neutral variants chosen at random in generation 3 (Fig. S1); thermostability and initial rates of neutral variants from generation 3 (Fig. S2); SDS-PAGE of PaDa-I and neutral variants from culture broth (Fig. S3); purification of UPO variants (Fig. S4); thermostability and activity in the presence of organic cosolvents (Fig. S5); amino acid mutations of neutral clones from generation 8 (Table S1).

  PDF, 577K
