# Supplementary material for: Shuffling the Neutral Drift of Unspecific Peroxygenase in Saccharomyces cerevisiae
Source: Appl Environ Microbiol. 2018 Jul 17;84(15):e00808-18. doi: 10.1128/AEM.00808-18 (PMC6052263; doi:10.1128/AEM.00808-18)
Supplement: Supplemental material [file AEM.00808-18_zam015188636s1.pdf]

Supplementary material for:

Shuffling the neutral drift of unspecific peroxygenase in *Saccharomyces cerevisiae*

Javier Martin-Diaz[1], Carmen Paret[1], Eva García-Ruiz[2], Patricia Molina-Espeja[1] and Miguel Alcalde[1]*

[1]Department of Biocatalysis, Institute of Catalysis, CSIC, 28049 Madrid, Spain.

[2]Manchester Institute of Biotechnology, The University of Manchester, 131 Princess Street, Manchester M1 7DN, UK.

*Address correspondence to Miguel Alcalde: malcalde@icp.csic.es.

Key words: unspecific peroxygenase, neutral genetic drift, *in vivo* DNA shuffling, *Saccharomyces cerevisiae*, directed evolution.

Running title: engineering peroxygenases by neutral drift and DNA shuffling.

1-EPGLPPGPLE**N**SS**A**KLVNDE**A**HPW**K**PLRPGDIRGPCPGLNTLASHGYLPR -50

51-NGVATPAQII**N**AVQEGFNFDNQAAIFATYAAHLVDGN**L**ITDLLSIGRKTR -100

101-LTGP**D**PPPPA**SV**GGL**N**EHGTFEGDASMTRGDAFFGNNHDFNETLFEQLVD-150

151-YSNRFGGGKYNLTVAGELRFKRIQDSIA**T**NPNFSFVDFRFFTAYGET**T**FP-200

201-ANLFVDGRRDDGQLDMDAAR**S**FFQFSRMPDDFFRAPSPRSGTGVEV**V**VQA-250

251-HPMQPGRNVGKINSYTVDPTSSDFSTPCLMYE**K**FVN**I***TVKSLYPNPT**V**QL-300

301-R**K**ALNTNLDFLFQGVAAG**C**TQVFPYGRD-329

VARIANTS

1	●	26	●
4	●	27	●
10	●	30	●
15	●	33	●
18	●	34	●
20	●	39	●
21	●		

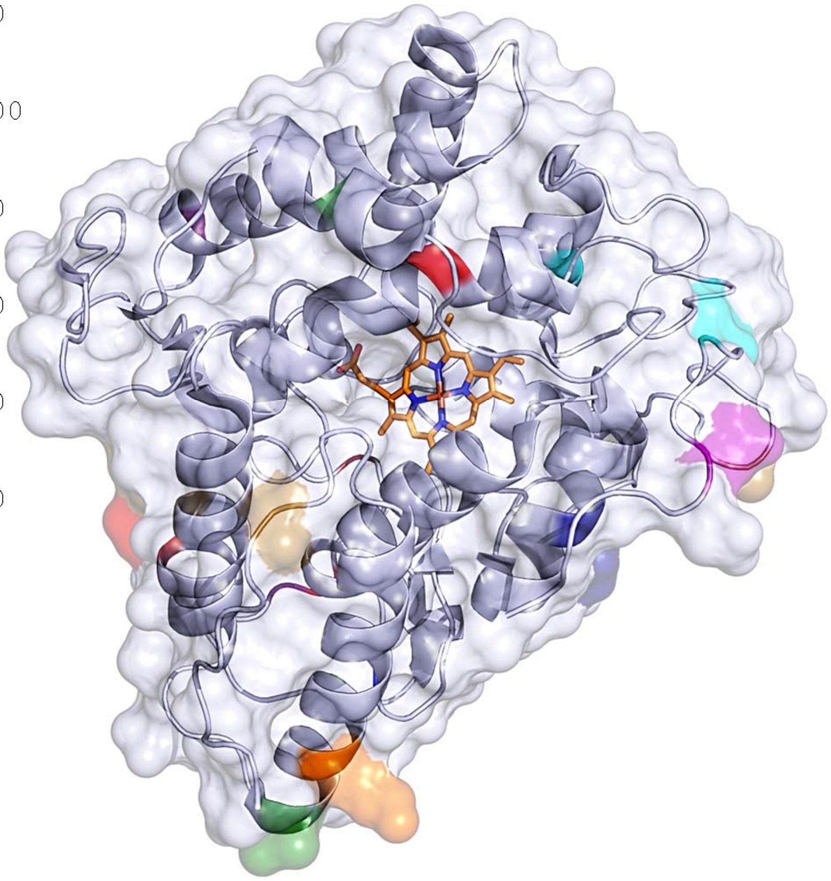

Fig. S1. Mutation of neutral variants chosen at random in generation 3. Mutations are highlighted in different colors relative to the variant number. The asterisk at position Ile287 of the fasta file is a double mutation for variant 1 (I287V) and variant 26 (I287M). Mutations mapped in the *A. aegerita* UPO crystal structure (PDB accession number 2YOR).

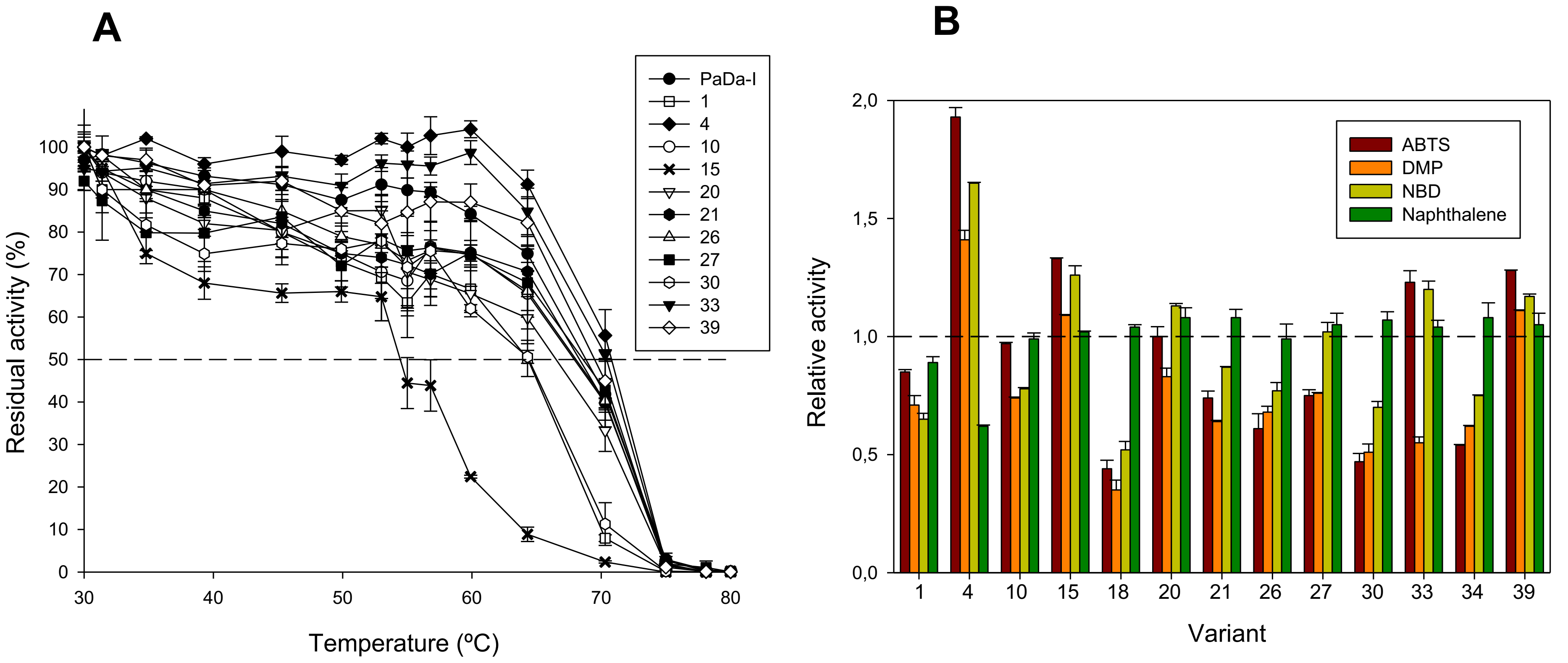

Fig. S2. Thermostability and initial rates of neutral variants from generation 3. (A) Thermostability (T_{50}) of the parental PaDa-I and the neutral variants. **(B)** Initial turnover rates for ABTS, DMP, NBD and naphthalene relative to that of the parental PaDa-I (dashed line). Each point represents the mean and standard deviation of 3 independent experiment from supernatant preparations as described in Material and Methods section.

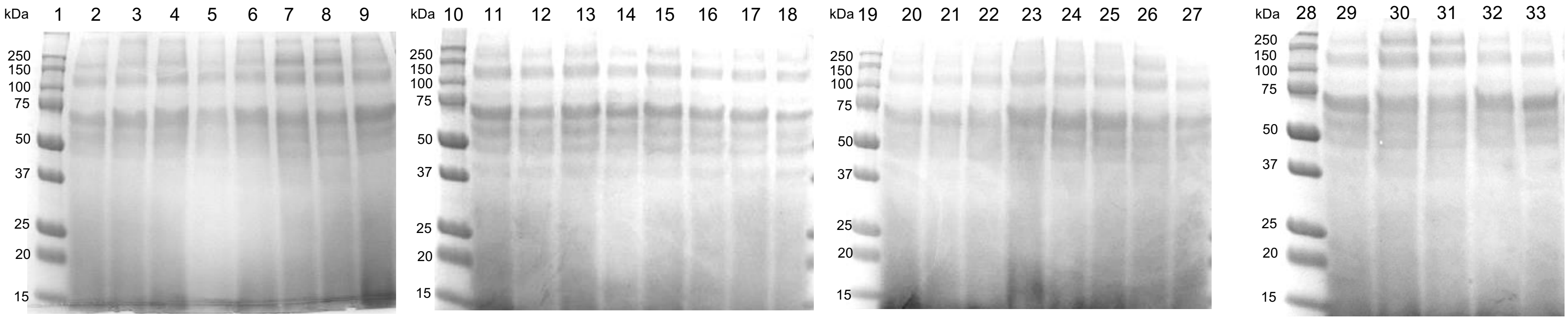

Fig.S3. SDS-PAGE of PaDa-I and neutral variants from culture broth: 12 % SDS-PAGE stained with SeeBand protein staining solution (Gene Bio-Application Ltd, Israel). Lanes 1, 10, 19 and 28, protein markers; 2, 11, 20 and 29, PaDa-I; 3, clone 4.1; 4, clone 4.2; 5, clone 4.4; 6, clone 4.6; 7, clone 4.7; 8, clone 4.8; 9, clone 4.9; 12, clone 5.9; 13, clone 6.1; 14, clone 6.6; 15, clone 7.1; 16, clone 7.2; 17, clone 7.6, 18, clone 11.1; 21, clone 11.9; 22, clone13.5; 23, clone 16.3; 24, clone 16.5; 25, clone 16.6; 26, clone 19.6; 27, clone 20.4; 30, clone 21.8; 31, clone 25.1; 32, clone 25.2; 33, clone 25.6.

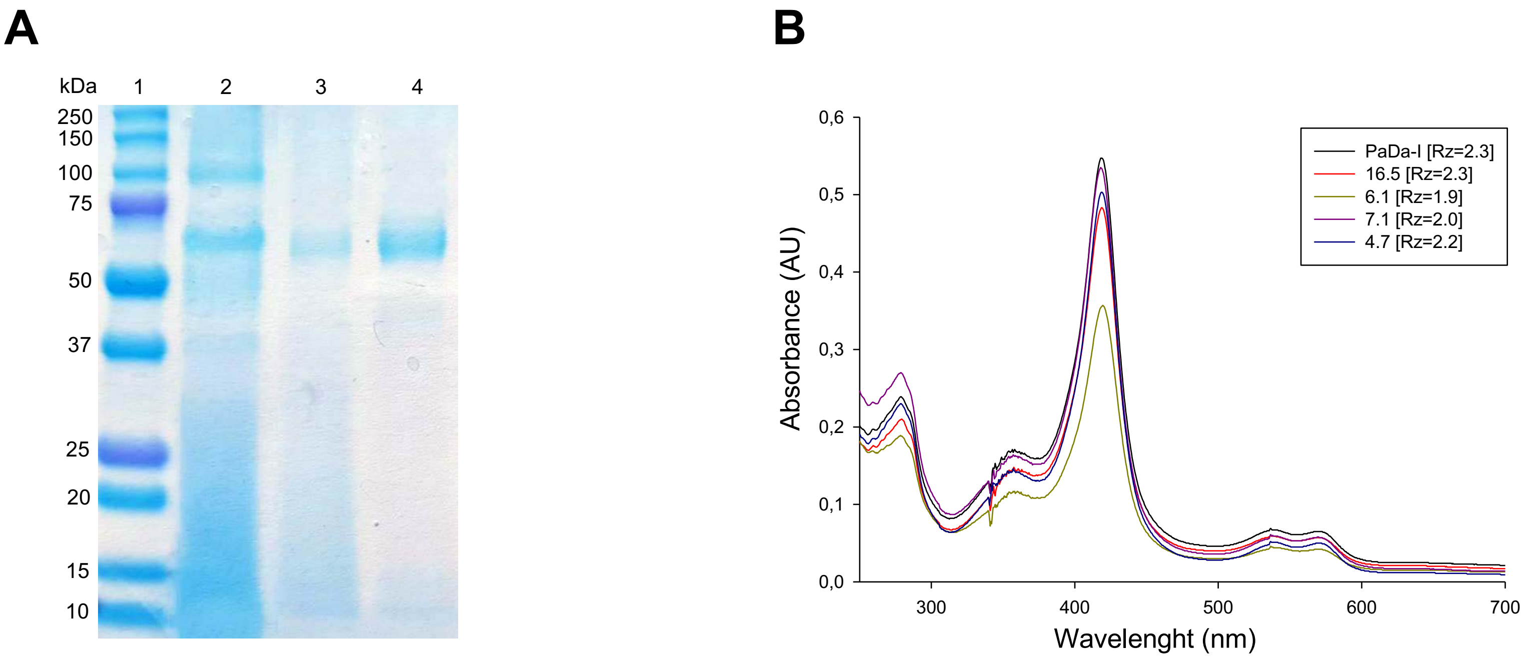

C

PaDa-I mutant	Total activity (U/mL)	[Protein] (mg/mL)	Specific activity (U/mg)	Purification factor
Culture broth	3.5	0.22	15.9	1
Cation exchange HiTrap SP FF	51	1.04	49.1	3.1
Anion exchange MonoQ	3634	4.78	760	47.8

Fig.S4. Purification of UPO variants: (A) 12 % SDS-PAGE stained with SeeBand protein staining solution (Gene Bio-Application Ltd, Israel). Lane 1 protein marker; 2, PaDa-I culture broth; 3, after cation exchange; 4, after anion exchange; **(B)** Spectroscopic analysis of purified parental PaDa-I and purified neutrally evolved UPO mutants with their corresponding Rz values; **(C)** Purification table for PaDa-I parental type.

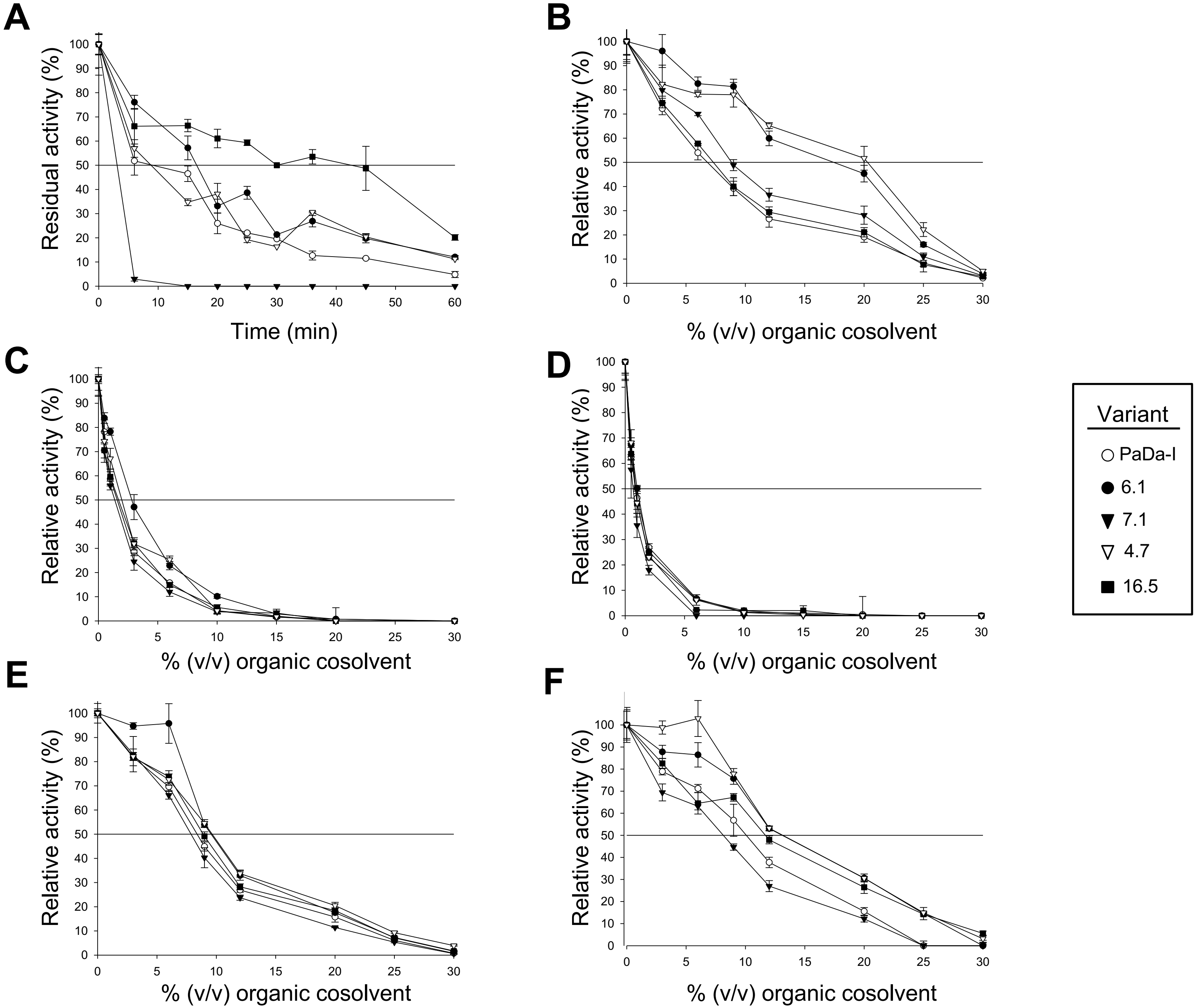

Fig. S5. **Thermostability and activity in the presence of organic co-solvents**. **(A)** Kinetic thermostability of the purified parental PaDa-I and neutral variants ($t_{1/2}$ measured at 63 ºC). **(B-D)** Activity at different concentrations (v/v) of organic co-solvents: **(B)** acetonitrile; **(C)** DMSO; **(D)** ethanol; (**E**) methanol; and (**F**) acetone. Each point represents the mean and standard deviation of 3 independent experiments (see also **Table 1**).

Table S1. Amino acid mutations of neutral clones from generation 8.

	PaDa-I	4.1	4.2	4.4	4.6	4.7	4.8	4.9	5.9	6.1	6.6	7.1	7.2	7.6	11.1	11.9	13.5	16.3	16.5	16.6	19.6	20.4	21.8	25.1	25.2	25.6
14	$_{GCA}A$																							T_{ACA}		
27	$_{CTT}L$																				I_{ATT}					
57	$_{GCG}A$			G_{GGG}																						
60	$_{ATA}I$																									V_{GTA}
61	$_{AAC}N$																S_{AGC}									
84	$_{GTG}V$																			A_{GCG}						
88	$_{CTC}L$																		P_{CCC}							
119	$_{GGC}G$											S_{AGC}														
130	$_{GGT}G$													S_{AGT}												
148	$_{TTG}L$				M_{ATG}																					
154	$_{CGA}R$							Q_{CAA}																		
186	$_{GTT}V$																			A_{GCT}						
188	$_{TTT}F$	L_{CTT}																L_{CTT}								
191	$_{TTT}F$	L_{CTT}								L_{CTT}						L_{CTT}										
197	$_{ACC}T$								A_{GCC}																	
198	$_{ACC}T$			A_{GCC}							A_{GCC}						A_{GCC}									
215	$_{GAT}D$																				E_{GAA}					
226	$_{AGC}S$		G_{GGC}				G_{GGC}		G_{GGC}	G_{GGC}			G_{GGC}												G_{GGC}	
233	$_{TTC}F$			Y_{TAC}							Y_{TAC}															
242	$_{ACA}T$																				S_{TCA}					
249	$_{CAG}Q$																		R_{CGG}							
254	$_{CAG}Q$									R_{CGG}					R_{CGG}				R_{CGG}							
270	$_{ACA}T$																					A_{GCA}				
272	$_{TCT}S$					P_{CCT}																				
280	$_{ATG}M$								L_{CTG}																	
290	$_{AAG}K$																					R_{AGG}	R_{AGG}			
317	$_{GCT}A$					D_{GAT}																				

Silent mutations are not included.
